# Spatio-temporal extension in site of origin for cortical calretinin neurons in primates

**DOI:** 10.3389/fnana.2014.00050

**Published:** 2014-06-26

**Authors:** Ana Hladnik, Domagoj Džaja, Sanja Darmopil, Nataša Jovanov-Milošević, Zdravko Petanjek

**Affiliations:** ^1^Department of Anatomy and Clinical Anatomy, School of Medicine, University of ZagrebZagreb, Croatia; ^2^Department of Neuroscience, Croatian Institute for Brain Research, School of Medicine, University of ZagrebZagreb, Croatia

**Keywords:** interneurons, calretinin, ganglionic eminence, ventricular zone, GABA, epilepsy

## Abstract

The vast majority of cortical GABAergic neurons can be defined by parvalbumin, somatostatin or calretinin expression. In most mammalians, parvalbumin and somatostatin interneurons have constant proportions, each representing 5–7% of the total neuron number. In contrast, there is a threefold increase in the proportion of calretinin interneurons, which do not exceed 4% in rodents and reach 12% in higher order areas of primate cerebral cortex. In rodents, almost all parvalbumin and somatostatin interneurons originate from the medial part of the subpallial proliferative structure, the ganglionic eminence (GE), while almost all calretinin interneurons originate from its caudal part. The spatial pattern of cortical GABAergic neurons origin from the GE is preserved in the monkey and human brain. However, it could be expected that the evolution is changing developmental rules to enable considerable expansion of calretinin interneuron population. During the early fetal period in primates, cortical GABAergic neurons are almost entirely generated in the subpallium, as in rodents. Already at that time, the primate caudal ganglionic eminence (CGE) shows a relative increase in size and production of calretinin interneurons. During the second trimester of gestation, that is the main neurogenetic stage in primates without clear correlates found in rodents, the pallial production of cortical GABAergic neurons together with the extended persistence of the GE is observed. We propose that the CGE could be the main source of calretinin interneurons for the posterior and lateral cortical regions, but not for the frontal cortex. The associative granular frontal cortex represents around one third of the cortical surface and contains almost half of cortical calretinin interneurons. The majority of calretinin interneurons destined for the frontal cortex could be generated in the pallium, especially in the newly evolved outer subventricular zone that becomes the main pool of cortical progenitors.

## INTRODUCTION

Neurons of the mammalian neocortical network can be roughly divided in glutamatergic excitatory and GABAergic inhibitory neuron population (Nieuwenhuys, 1994; DeFelipe et al., 2002, 2013). The first class, often referred as pyramidal neurons, is characterized by dendrites covered with spines and by long axon that projects away from pial surface and travels through white matter to target other cortical (corticocortical projections) or subcortical regions (corticofugal projections). Nearly all pyramidal neurons also send recurrent collateral branches which end in the local cortex (DeFelipe and Farinas, 1992; Spruston, 2008; Thomson, 2010; Zaitsev et al., 2012). During development, some features common to the typical pyramidal neurons are transformed by reduction, but analogy with the dendritic morphology of typical pyramidal cell is obvious. These neurons can be defined as modified pyramidal neurons (Braak and Braak, 1985). The spiny stellate cells in layer IV of the primary sensory areas are glutamatergic neurons that retract their output axon branch during development but retain only spiny dendrites and an intracortical axonal arbor (Vercelli et al., 1992; Nieuwenhuys, 1994; Markram et al., 2004). Glutamatergic cortical neurons are usually defined as principal, while in mammals they are (depending on region and species) 3–5 times more numerous than those synthesizing GABA. Still less abundant, cortical GABAergic neurons form extremely heterogeneous population with regard to their connectivity, molecular features, and physiological properties (DeFelipe et al., 2013). The vast majority of GABAergic neurons has axon branching only inside the cortex and are therefore defined as local circuit neurons (interneurons). A small amount of cortical GABAergic neurons has axons turning into the white matter (Tomioka and Rockland, 2007; Melzer et al., 2012). They are remnants of the earliest functional fetal neocortical network (Kostovic and Rakic, 1990; Super and Uylings, 2001; Judas et al., 2013).

Cortical GABAergic neurons are internal modulators of cortical output that is achieved through direct and indirect control of principal neuron compartments (Jones, 1993). Some of the cortical GABAergic neurons subtypes are targeting the axon initial segment of principal neurons, some are making synapses mainly on cell bodies and proximal dendrites, whereas others are targeting more distal parts of principal neurons dendritic tree (DeFelipe et al., 2013). Interestingly, some cortical GABAergic neurons preferentially target other cortical GABAergic neurons (Caputi et al., 2009).

Each of the main cortical GABAergic neurons group defined on the basis of efferent connectivity is characterized by remarkable physiological diversity and could be divided into distinct subtypes (Markram et al., 2004; Ascoli et al., 2008; DeFelipe et al., 2013). Additional heterogenity is showed by expression of molecular markers. Nevertheless, molecular classification is suitable to study regional and species differences in laminar distribution and site of origin for different cortical GABAergic neurons subtypes. High expression of three calcium-binding proteins, parvalbumin, calbindin and calretinin is present only in cortical GABAergic neurons, and not in neurons expressing glutamate. More than 80% of all cortical GABAergic neurons express one of these three calcium-binding proteins without significant overlap, making these molecules a good marker for identification of different cortical GABAergic neuron subpopulations (Van Brederode et al., 1990; Kubota et al., 1994; Yan et al., 1995; Gonchar and Burkhalter, 1997; Tamamaki et al., 2003; Gonchar et al., 2007; Burkhalter, 2008; Uematsu et al., 2008; Xu et al., 2010). It was found that calbindin-positive cells also express somatostatin (Rogers, 1992; Kubota et al., 1994; Gonchar and Burkhalter, 1997; Kawaguchi and Kubota, 1997). However, around one third of somatostatin-positive cells do not express detectable level of calbindin. They represent a larger proportion of cortical GABAergic neurons and also include neuronal nitric oxide synthase (nNOS) expressing cells, as well as neuropeptide Y positive but calbindin-negative neurons. Therefore, the neuropeptide somatostatin was shown to be a more eligible marker than calbindin, enclosing a larger subset of cortical GABAergic neurons without overlap to other two major subpopulations. Neurochemical features of interneurons can be coupled with their synaptic targets. The parvalbumin-expressing cells are mainly targeting soma and perisomatic region, including axon initial segment of principal cells. The calretinin-expressing cells are targeting proximal parts of dendritic tree, whereas somatostatin-expressing are targeting the distal dendritic regions of pyramidal neurons.

**FIGURE 1:**
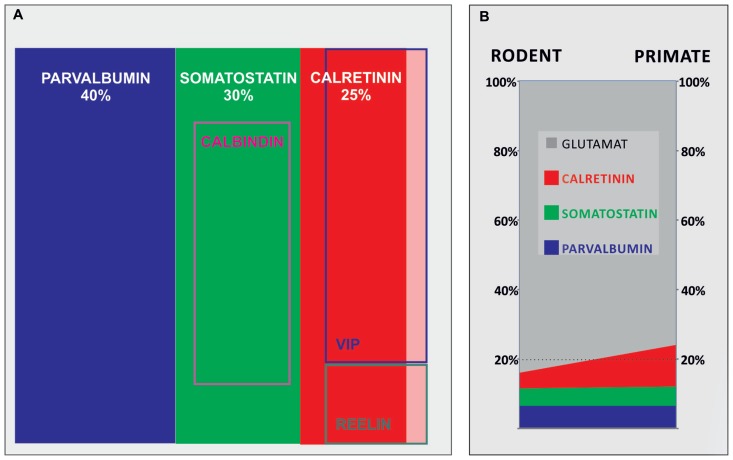
**A contribution of main cortical GABAergic neuron subpopulations to total number of GABAergic neurons in rodents and their contribution to total number of neurons in rodents and primates. (A)** Demonstrates contributions of different subpopulations of GABAergic neurons in rodents, showing that parvalbumin, somatostatin, and calretinin comprise more than 90% of all GABAergic neurons. **(B)** Demonstrates 50% of increase of the contribution of cortical GABAergic neurons from rodents (≈16%) to primates (≈24%), which can be attributed to threefold increase in number of calretinin neurons (shown in red; from ≈4% in rodents to ≈12% of the total neuron number in primates).

Recent data suggest that the most numerous cortical GABAergic neurons subpopulation in rodent is parvalbumin subpopulation. The parvalbumin-expressing neurons account for about 40% of total cortical GABAergic neurons population in rodents, around 30% of cortical GABAergic neurons are somatostatin-expressing and 25% are calretinin-expressing neurons (Gonchar et al., 2007; Uematsu et al., 2008; Xu et al., 2010). Part of the calretinin neurons co-express vasoactive intestinal peptide or reelin. However, some cells express vasoactive intestinal peptide or reelin only and represent around 5% of the total cortical GABAergic neurons (Burkhalter, 2008; Ma et al., 2013; **Figure 1**). In addition, recent data suggest that population of calretinin, vasoactive intestinal peptide and reelin-expressing cells in rodents can be identified by expression of the serotonin receptor 5HT3aR. Heterogeneous 5HT3aR-expressing population comprise approximately 30% of all interneurons, and together with parvalbumin- and somatostatin-expressing population account for nearly 100% of all mouse cortical GABAergic neurons (Rudy et al., 2011). As recently discovered, 5HT3aR population has to be further characterized.

## PRONOUNCED INCREASE IN PROPORTION OF CALRETININ NEURONS DISTINGUISH GABAergic CORTICAL NETWORK OF PRIMATES

Numerous studies analyzing laminar distribution and density of cortical GABAergic neurons subtypes were performed in various species (Hendry et al., 1987; Van Brederode et al., 1990; Rogers, 1992; Beaulieu, 1993; Kubota et al., 1994; Micheva and Beaulieu, 1995; Yan et al., 1995; Del Rio and DeFelipe, 1996; Gabbott and Bacon, 1996a, b; Del Rio and DeFelipe, 1997; Gabbott et al., 1997a, b; Gonchar and Burkhalter, 1997; Tamamaki et al., 2003; Zaitsev et al., 2005; Gonchar et al., 2007; Sherwood et al., 2007; Tomioka and Rockland, 2007; Uematsu et al., 2008; Xu et al., 2010; Barinka et al., 2012). Due to different methodological approaches, it is very difficult to perform consistent comparative analysis of regional, laminar and species differences that surely exist. Intention to make collective conclusion about proportion of cortical GABAergic neurons and their subtypes in animals of particular order is therefore oversimplified (Burkhalter, 2008). Nevertheless, reports analyzing multiple regions suggest considerable consistency in the proportion and laminar distribution between various cortical regions inside the species of the same order (Tamamaki et al., 2003; Xu et al., 2010). It seems that even the main pattern of laminar and regional distribution of different cortical GABAergic neurons subtypes is highly conserved through mammalian evolution. However, there is no proportional increase in number of all cortical GABAergic neurons subtypes.

The vast majority of studies performed in rodents demonstrated that GABAergic neurons represent less than 20% of cortical neurons (Beaulieu, 1993; Micheva and Beaulieu, 1995; Gabbott et al., 1997a; Tamamaki et al., 2003). Parvalbumin is the most dominant population, whereas the calretinin is the less numerous (**Figure 1A**). Most of the studies suggest that in the human, where higher order associative areas comprise at least 50% of the neocortical surface, calretinin becomes the dominant population representing almost 50% of cortical GABAergic neurons (Conde et al., 1994; Gabbott and Bacon, 1996b; Zaitsev et al., 2005; Barinka and Druga, 2010). The number of GABAergic neurons increases more than is the case for principal neurons during mammalian evolution, so they exceed 20% of all cortical neurons in the primate neocortex (Hendry et al., 1987; Del Rio and DeFelipe, 1996; Gabbott and Bacon, 1996a, b; Jones, 2009). The proportion of cortical GABAergic neurons seems to increase for about 50% when primates (where cortical GABAergic neurons represent around 24% of total neuron number) are compared to rodents (where cortical GABAergic neurons represent around 16% of total neuron number). In parallel with increase of total neuron number, the number of parvalbumin- and somatostatin-expressing neurons increases linearly. Each of these subtypes represents between 5 and 7% of neurons in both, rodent and primate species. This would imply that increase in proportion of cortical GABAergic neurons is principally related to calretinin subtype which number increases exponentially, representing about 4% of total number of neurons in rodents and about 12% in primates (**Figure 1B**).

**FIGURE 2:**
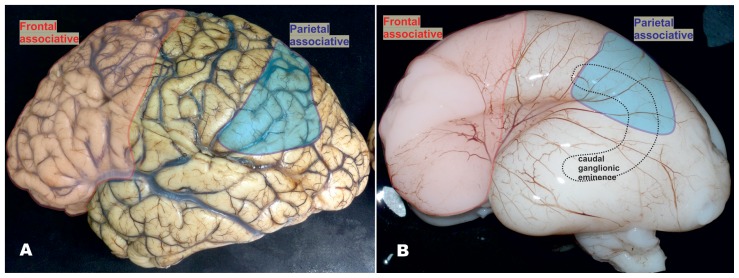
**Mature and fetal human brain around mid-gestation, showing that higher order multimodal areas enclose the frontal granular cortex and majority of parietal lobe (excluding postcentral gyrus). (A)** In the human brain, frontal granular cortex occupies 80% of the frontal lobe without clear correlate in mouse. In the humans, this cortex represents almost one third (red) of total cortical surface, as well as 80% of multimodal associative areas including associative areas of the parietal cortex (blue). In the higher order associative areas located in the frontal (red) and parietal lobe (blue), calretinin-expressing neurons represent almost 50% of GABAergic neuron population and more than 10% of total neuron population. Due to presumably smaller proportion of calretinin neurons in temporal and occipital cortices (Ma et al., 2013) and taking into the consideration the size of frontal granular cortex, we assumed that almost half of cortical calretinin neurons are located in the frontal lobe. **(B)** The proportions in the size of higher order associative cortices are already established around mid-gestation. In the recent study, Ma et al. (2013) concluded that vast majority of calretinin neurons in human originate from the caudal ganglionic eminence (CGE, dashed line showed a contour of its position) and that there is no abundant production of GABAergic neurons in the pallial proliferative zones as it was suggested before (Letinic et al., 2002). The anatomical relations between anteriorly located prefrontal cortex (that comprises almost half of cortical calretinin neurons) and postero-laterally located CGE (as a main source of calretinin neurons) do not concur with this presumption (see **Figure 5B**). We suggest that presumption of Ma et al. (2013) can be correct in humans for all cortical regions only during early fetal stage, and later on for the all lobes excluding frontal cortex (Al-Jaberi et al., 2013).

We expect that such a disproportional increase in number of one GABAergic neurons subtype will provoke significant changes in neuronal network organization and substantially different modes of signal processing (Burkhalter, 2008). The connection between exponentially increased number of calretinin neurons through human associative areas and tremendous increase in cognitive capability is speculative (Forbes and Grafman, 2010), but apparent (**Figure 2**). The majority of neurological and psychiatric disorders that involve cortical pathology are suggested to have some level of disorganization in GABAergic network (Friocourt and Parnavelas, 2011; Marin, 2012). Most of these disorganizations have developmental origin. While some disorders can be found only in humans, such as schizophrenia and autism, other disorders (e.g., epilepsy) have in humans more complex and specific symptomatology. Therefore, it is important to understand human-specific features in organization and development of cortical GABAergic network, especially for those showing main differences, as is calretinin neuron population. We suggest that evolution is partially changing rules about origin of this neuron subtypes.

## GE IS A MAIN SOURCE OF RODENT CORTICAL GABAergic NEURONS AND EARLY FETAL PRIMATE CORTICAL GABAergic NEURONS

**FIGURE 3:**
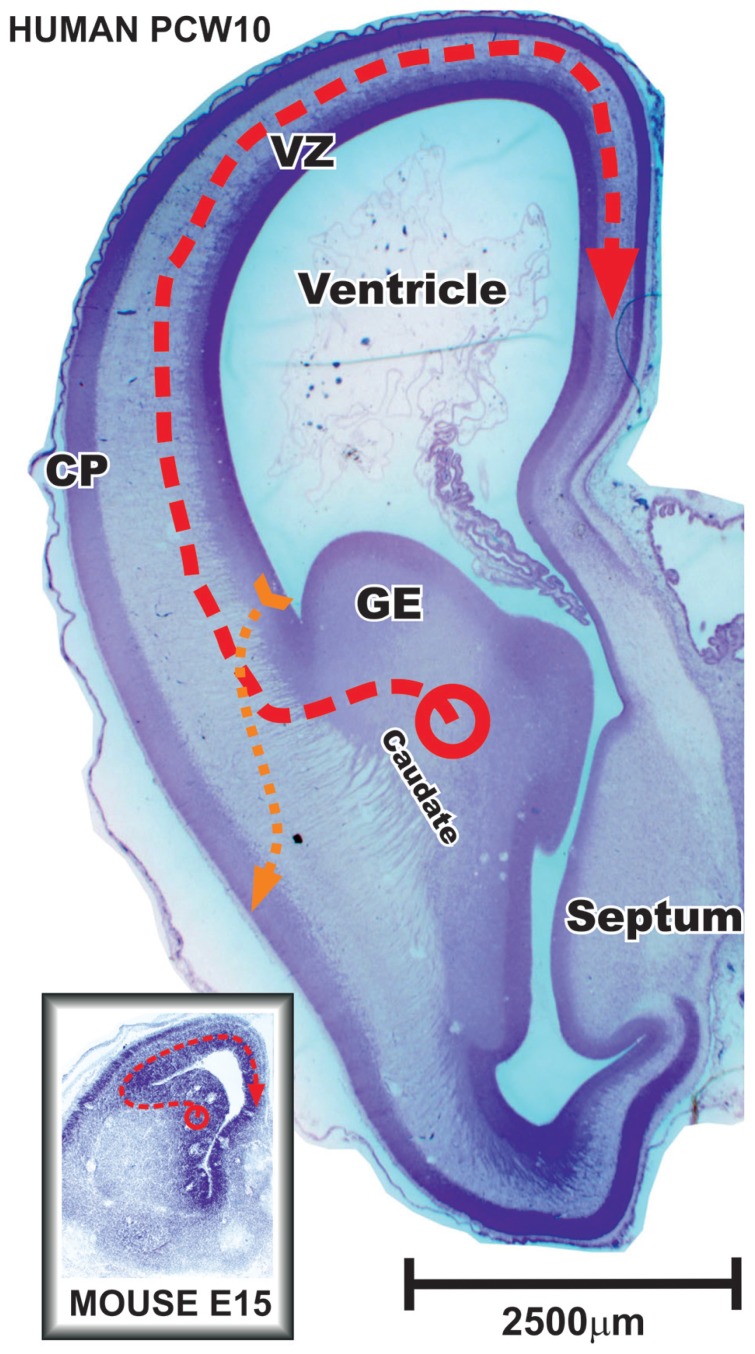
**Frontal section through the anterior part of the fetal human and mouse brain during early stages of cortical neurogenesis**. In primates during the early fetal period and in rodents during whole neurogenesis vast majority of cortical plate (CP) GABAergic neurons are produced in the ganglionic eminence (GE) and migrate tangentially into the cerebral cortex (red dashed line). Note that neurons tangentially migrating to the most distant positions in the mouse brain might pass the same distance as certain radially migrating glutamatergic principal cells in human brain (yellow dashed line) that originate in the pallial ventricular and subventricular zone (VZ). During middle trimester of gestation, when majority of cortical neurons are born, discrepancy in scaling between human and mouse is much more pronounced. Modified from Petanjek et al. (2009b).

During corticogenesis in mouse, from embryonic day (E) 11 to E19 vast majority, if not all cortical GABAergic neurons originate from moleculary and morphologically distinct regions of ventral telencephalon (Flames et al., 2007; Gelman and Marin, 2010) and migrate tangentially into the cerebral cortex (Van Eden et al., 1989; Parnavelas et al., 2002; Faux et al., 2010; Hernandez-Miranda et al., 2010; Antypa et al., 2011; **Figure 3**). A primary source is the medial ganglionic eminence (MGE), producing approximately 50–60% of the cortical GABAergic neurons with a peak of proliferation around E12–E13 (Wichterle et al., 2001; Wonders and Anderson, 2006; Butt et al., 2007; Gelman and Marin, 2010). The MGE is defined by the expression of homeobox transcription factor Nkx2.1 (Sussel et al., 1999; Butt et al., 2008). The parvalbumin-expressing subpopulation originate from Nkx2.1 progenitors in the ventral part of MGE, while the Nkx6.2 co-expressing progenitors from the dorsal part of MGE produce somatostatin subpopulation of cortical GABAergic neurons (Flames et al., 2007; Fogarty et al., 2007; Wonders et al., 2008). After leaving the MGE, migratory cortical GABAergic neurons downregulate Nkx2.1 but maintain Sox6 expression necessary for their normal positioning and maturation (Batista-Brito et al., 2009).

The second greatest source of GABAergic neurons progenitors is the caudal ganglionic eminence (CGE), producing in mouse about 30% of all cortical GABAergic neurons with a later peak of neurogenesis, around E16.5 (Miyoshi et al., 2010). Progenitors from the CGE express orphan nuclear factors COUP-TF I/II (Kanatani et al., 2008) and generate diverse subtypes of cortical GABAergic neurons that preferentially occupy superficial cortical layers (Miyoshi et al., 2010). The CGE-derived interneurons include two large groups: bipolar interneurons that express calretinin and/or vasoactive intestinal peptide (Xu et al., 2004; Butt et al., 2005; Wonders and Anderson, 2006), as well as neurogliaform reelin-positive cells (Miyoshi et al., 2010).

The third source giving nearly 10% of GABAergic neurons in murine cerebral cortex is the preoptic area. Different lineages within the preoptic area, such as Nkx5.1 and Dbx1 lineage, generate a great diversity of cortical GABAergic neurons (Gelman et al., 2011).

The remaining major part, the lateral ganglionic eminence (LGE), is a source of striatal GABA projection neurons, olfactory bulb and amygdala interneurons (Corbin et al., 2008). The LGE serves as a migratory route for interneurons derived from adjacent MGE (Wichterle et al., 2001) and in smaller extent from the CGE (Miyoshi et al., 2010). In contrast to the largest part of the LGE, the most dorsal part (dLGE) that is adjacent to proliferative zone of the dorsal telencephalon is characterized by strong expression of the zinc finger transcription factor Sp8 and produces calretinin neurons for olfactory bulb and amygdala. In the mouse, only a small fraction of calretinin-expressing cortical GABAergic neurons is found to be derived from progenitors within the dLGE. In the humans, it is suggested that Sp8 progenitors from the dLGE give more important contribution to calretinin-expressing cortical GABAergic neurons due to smaller needs for olfactory bulb neurons (Ma et al., 2013).

Recent studies showed conserved pattern underlying the expression of transcription factors in the subpallium of monkey and human fetuses (Hansen et al., 2010; Ma et al., 2013; Pauly et al., 2013). In addition, pattern of origin for main subpopulations of cortical GABAergic neurons derived in the subpallium is also conserved. Sox6-expressing progenitors within cells leaving the MGE give somatostatin and parvalbumin subpopulations whereas COUP-TFII and/or Sp8-expressing cells leaving the CGE and dLGE contribute to cortical calretinin subpopulation (Ma et al., 2013). Although, a considerable increase in proportion and number of calretinin neurons in human cortex requires expanded pool of progenitors. Several studies showed that a novel pool of GABAergic neuron progenitors appears in the pallial (cortical) proliferative zones (Jakovcevski et al., 2011; Al-Jaberi et al., 2013) and becomes one of the main sources for cortical GABAergic neurons after the early fetal period (Letinic et al., 2002; Rakic and Zecevic, 2003; Petanjek et al., 2009a, b). Even though evidence for early fetal production of cortical GABAergic neurons is strong (Petanjek et al., 2009a; Jakovcevski et al., 2011; Al-Jaberi et al., 2013), most of available data suggest that during the early fetal period in primates [E47–55 in monkey and from 8 to 13 postconceptional weeks (pcw) in human] the majority of cortical GABAergic neurons is generated in the GE (**Figure 3**). Laminar and cellular organization during the early fetal stage in primates (Ip et al., 2010) corresponds to whole neurogenetic stage of rodents (Rakic, 2009). Later on during development, primate proliferative compartments reveal novel features of organization and do not have clear similarities with rodents (see the next chapter).

However, already during the early fetal period a largely increased pool of GE progenitors was observed in both monkey and human (Hansen et al., 2013). Previously unknown type of non-epithelial neural stem cell lacking radial fibers populated massively expanded subventricular zone (SVZ) of the GE. The MGE exhibited unique patterns of progenitor cell organization and clustering. The important production of cortical interneurons in the dLGE was observed and CGE generated a higher proportion of cortical GABAergic neurons than in rodent. The increased early production of cortical GABAergic neurons within this expanded compartment undoubtedly contributes to considerable higher proportion of calretinin neurons. But, considerably more cortical neurons are generated after the early fetal period when the peak of neurogenesis occurs (Rakic, 2002). Even if increased pool in the CGE continues with proportionally higher production of calretinin neurons later on during second trimester of gestation, we do not believe that it could account for increased need of calretinin neurons in all cortical regions of the human brain.

## PALLIAL PRODUCTION OF CORTICAL GABAergic NEURONS IN PRIMATES DURING SECOND TRIMESTER OF GESTATION

Several reports suggested that during the second trimester of gestation in primates (E64–E75 in monkey and 15–24 pcw in humans) neocortical proliferative zones produce a substantial percentage of cortical GABAergic neurons (Letinic et al., 2002; Rakic and Zecevic, 2003; Petanjek et al., 2009b; Yu and Zecevic, 2011; Zecevic et al., 2011). Reports from rodents showed that the dorsal proliferative zones account for only a very small fraction (if any) of cortical GABAergic neurons present at maturity (Xu et al., 2004; Molnar et al., 2006; Wonders and Anderson, 2006). From present studies performed in non-primate species, the notable fraction of pallialy derived cortical GABAergic neurons was observed in ferret, an animal that has more convoluted cortex with enlarged proportion of cortical projection neurons in layers II and III. In ferret up to 5% of layer II and III cortical GABAergic neurons originate from the pallium (Anderson et al., 2002).

**FIGURE 4:**
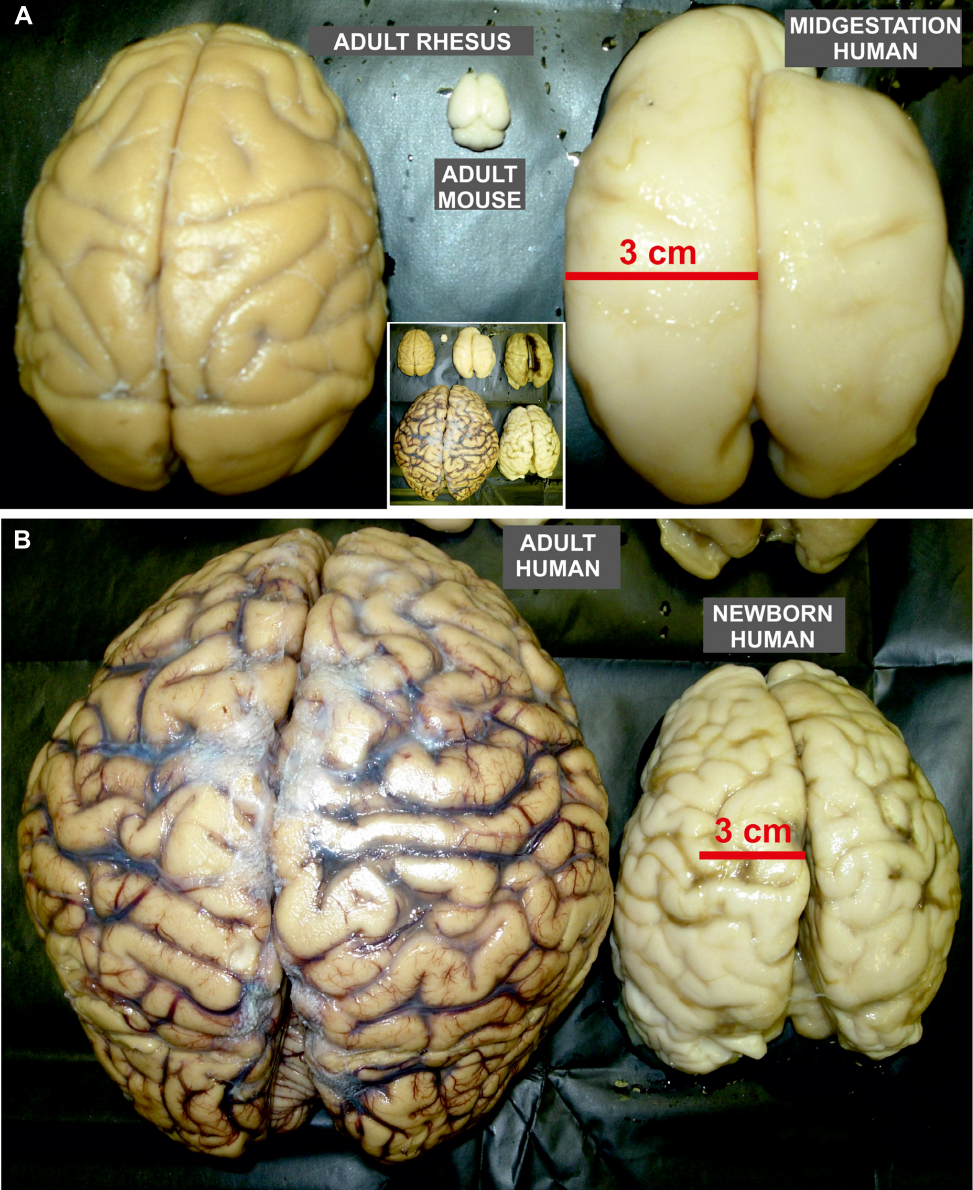
**Size proportion of mature rodent and non-human primate brain as well as developing and mature human brains**. Dorsal view of adult mouse **(A)**, rhesus monkey **(A)**, and human brain **(B)**, as well as human fetal brain around mid-gestation **(A)** and at term **(B)**. **(A)** The size of human fetal brain already at mid-gestation has reached the size of the adult rhesus monkey brain. Nevertheless, adult rhesus monkey brain is almost 100 times larger than brain of adult mouse. **(B)** The adult human brain is around 3–4 times larger than newborn brain which size reaches the size of adult chimpanzee brain. Note that the pattern of gyrification in human newborn brain is close to the one observed in adult. The inlet in the middle of the figure is the integrative photo of all brains shown in **A** and **B** that demonstrate their actual proportions (the right brain in the upper row of insertion is from fetus at the beginning of third trimester of gestation).

The proportion of dorsally derived cortical GABAergic neurons dramatically increases in a larger human brain (**Figure 4**) in parallel with the demand of upper cortical layers (Rakic, 2009). Using retroviral labeling in organotypic slice cultures of the human fetal forebrain, Letinic et al. (2002) demonstrated the existence of two distinct lineages of cortical GABAergic neurons. The Dlx1/2-expressing lineage originates from the GE and accounts for 35% of cortical GABAergic neurons. The second lineage that expresses Dlx1/2 and Mash1 transcription factors is produced in the neocortical ventricular/subventricular zones (VZ/SVZ) at later stages (15–22 pcw) and represents around 65% of cortical GABAergic neurons. It was suggested that massive dorsal production of GABAergic neurons in larger brains might be an answer to facilitate migratory mechanisms and simplify migratory routes through exponentially expanding neocortex (Tan, 2002; Molnar et al., 2006). In those brains, migration from the GE at later developmental stage will become very complex and vulnerable.

**FIGURE 5:**
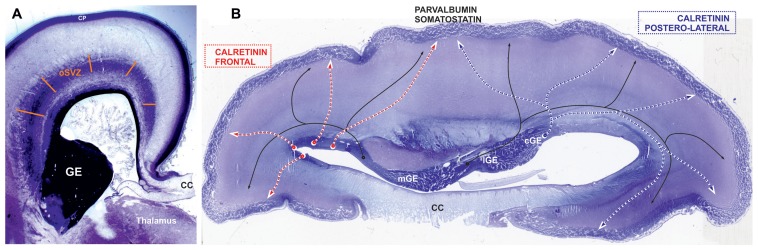
**Places of origin for different populations of GABAergic neurons**. Nissl-stained sections: **(A)** frontal section at the level of thalamus and **(B)** horizontal section through the body of corpus callosum (CC) at the end of middle trimester of gestation. **(A)** The section shows main proliferative compartment of the human pallium, the outer subventricular zone (oSVZ), that is the most plausible candidate for a major source of additional pool of progenitors of GABAergic neurons (Petanjek and Kostović, 2012). In favour of this view, already during the early fetal period human cortical progenitor cells in frontal lobe have capacity to generate inhibitory interneurons (Al-Jaberi et al., 2013). **(B)** In the mouse vast majority of parvalbumin and somatostatin (black line)-expressing GABAergic cortical plate (CP) neurons originate from the medial ganglionic eminence (mGE), while calretinin expressing originate from the caudal ganglionic eminence (CGE). The progenitors from lateral ganglionic eminence (lGE) give GABAergic neurons mainly for the subcortical structures. This spatial pattern is preserved during early trimester of gestation in primates, but with exponential increase in the pool of cGE progenitors (Hansen et al., 2013). We propose that during middle trimester of gestation, when majority of cortical neurons are produced, cGE remains the main source of cortical calretinin neurons (blue dashed line) for parietal, occipital, and temporal lobes (postero-lateral telencephalon), whereas the frontal lobe (red dashed line) is supplied with calretinin neurons from the oSVZ (see **Figure 2B**). Not only the anatomical relations (rather distant postion of frontal lobe from cGE) that do not concur with exclusive production of calretinin neurons from cGE (Ma et al., 2013), but also a threefold increase in proportion of calretinin neurons in the frontal cortex cannot be achieved through increase in pool and protracted production of progenitors in cGE.

Most likely, a main source of pallialy produced cortical GABAergic neurons is evolutionary new, outer proliferative compartment within the subventricular zone (oSVZ) that is not found in rodents (**Figure 5A**). The oSVZ becomes the main site of neuron production in primates, allowing huge expansion of upper cortical layers (Dehay and Kennedy, 2007; Bayatti et al., 2008; Clowry et al., 2010; Molnar and Clowry, 2012; Petanjek and Kostović, 2012). The oSVZ appears at the end of early fetal period and contains the vast majority of pallial progenitors during main stage of neurogenesis, the second trimester of gestation (E60–E90 in monkey, 15–24 pcw in humans). In holoprosencephaly, syndrome with severe striatal hypoplasia and atrophy of the GE, calretinin neurons are preserved, while there is a high decrease in number of other cortical GABAergic neurons subclasses (Fertuzinhos et al., 2009). This suggests that pallial GABAergic neuron progenitors mainly produce calretinin subpopulation. In favor of this conclusion, it has been showed that many of late-born dorsally derived interneurons are calretinin cells that also include double-bouquet cells abundant only in the upper cortical layers of the human neocortex (DeFelipe, 2011; Jakovcevski et al., 2011). In addition, recent *in vitro* study suggests that human, but not mouse Nkx2.1 expressing dorsal radial glial cells have the potential to generate cells of interneuronal lineage labeled with calretinin (Yu and Zecevic, 2011). Taking into consideration those findings, we would like to point that calculation of Letinić (suggesting 2/3 of cortical GABAergic neurons derived in the pallium) might be inaccurate due the fact that the dLGE and CGE are also important source for calretinin neurons. Therefore, if pallial proliferative zones produce mainly calretinin neurons, the amount of the dorsally derived cortical GABAergic neurons proposed by Letinic is overestimated and it would hardly exceed half of proposed amount (1/3 of cortical GABAergic neurons).

By labeling newborn neurons in slice culture and mapping of proliferating interneuron progenitors, as well as studying expression patterns of several key transcription factors in the developing human and monkey telencephalon, Hansen et al. (2013) and Ma et al. (2013) were not able to demonstrate extensive pallial production. They proposed that the majority of primate cortical GABAergic neurons originate from the GE. Protracted presence of proliferation in the primate GE during the second trimester of gestation and increased pool of calretinin progenitors in the CGE might explain increased proportion of calretinin neurons. However, it is difficult to imagine that this source can be sufficient to supply calretinin neurons of the human frontal cortex (**Figures 2B and 4**). The granular frontal cortex, that includes mainly higher order associative areas and occupies one third of human cortical surface (**Figure 2A**), is showing the most prominent evolutionary expansion without a correlation in rodents (Uylings and van Eden, 1990; Groenewegen and Uylings, 2000; Uylings et al., 2003; Rakic, 2009; Petanjek et al., 2011; Teffer and Semendeferi, 2012). In the regions abundant with cortico-cortical neurons, as are the higher associative region of the frontal and parietal cortex (Ma et al., 2013), calretinin neurons represent almost half of cortical GABAergic neuron population (Gabbott et al., 1997b). Therefore, we found unlikely that the CGE, even with expanded pool of progenitors and highly protracted neurogenesis, could be able to provide calretinin neurons if there is threefold increase in their proportion. In addition, newly evolved higher order associative areas are not in close range of the CGE, what logically assumes that production should occur more closely (**Figure 2B**). The primate dLGE, as a rostral extension of the CGE becomes in primate more cortical than subcortical proliferative pool (as is the case in rodent). While it is closer to the frontal lobe, it could primarily produce calretinin neurons for the frontal cortex. However, taking into account amount of progenitors in the dLGE and needs for calretinin neurons in the frontal cortex, it is hard to imagine that the dLGE could be their main source.

We propose that contradictory results observed in recent studies about cortical GABAergic neurons origin in primates, those suggesting abundant pallial production of cortical GABAergic neurons (Letinic et al., 2002; Rakic and Zecevic, 2003; Zecevic et al., 2005, 2011; Mo et al., 2007; Fertuzinhos et al., 2009; Petanjek et al., 2009a; Al-Jaberi et al., 2013) and those suggesting only minor dorsal production of cortical GABAergic neurons (Hansen et al., 2013; Ma et al., 2013), might be explained by regional variation in the site of calretinin neuron origin. Al-Jaberi et al. (2013) strongly suggest that human cortical progenitor cells in the frontal lobe have capacity to generate inhibitory interneurons. Therefore, in the primates, the CGE might be the main source for posterior and lateral regions of the cerebral cortex, whereas frontal lobe is supplied mainly by dorsally derived calretinin neurons (**Figure 5B**). While regional variations in cortical GABAergic neurons production were not analyzed in the studies performed until now, these hypotheses need further confirmation.

## PROTRACTED PRODUCTION OF CORTICAL GABAergic NEURONS FROM THE GE DURING THE LAST TRIMESTER OF GESTATION

If massive dorsal production of cortical GABAergic neurons in human is evolutionary answer to keep migratory routes shorter and simpler, one could expect that migration from the GE at later developmental stage will be exhausted. Comparing E55/E64 with E75 in monkey fetuses we could not qualitatively observe a decrease in amount of tangentially migrating GAD65 positive, prospective cortical GABAergic neurons arising from the GE (Petanjek et al., 2009a). There was also a preserved pool of GAD positive progenitors in the GE. In human fetuses at 24–26 pcw, extended stream of densely populated Golgi impregnated migratory like cells leaving GE was observed. Qualitative impression of amount in cells leaving GE together with its size suggests that peak of neurogenesis in the human GE is close to the end of second trimester of gestation (Petanjek et al., 2008). These data suggest that pallial production is not an answer to keep migratory routes shorter and simpler. Pallial production is most likely novel pool of progenitors that produces specific subtypes of cortical GABAergic neurons, such as the calretinin neurons.

BrDU studies in primates showed that the vast majority of cortical neurons are already born by the beginning of the second half of gestation (Rakic, 2002). Therefore, it is interesting that proliferation and migration of prospective GABAergic neurons from the GE are not declining at this period. In the recent study, Ma et al. (2013) observed numerous migratory like cells in the SVZ of the human neonatal brain what suggests that the GE as a proliferative compartment in human fetuses is preserved up to last trimester of gestation (Letinic and Kostovic, 1997). By evaluating human material of Zagreb neuroembryological collection (Judaš et al., 2011), we observed during the late fetal period many MAP-2 and calretinin-positive, non-radially oriented migratory like cells in the dorsal telencephalon, as in the stream leaving the GE (Hladnik et al., 2011, 2014). These preliminary observations are consistent with the hypothesis of protracted production of cortical GABAergic neurons. However, some reports suggest that cell proliferation is almost exhausted in VZ/SVZ of the GE by the last trimester of gestation (Zecevic et al., 2011). But if even neurogenetic potential in the GE decreases sharply during second half of gestation, additional 10–12 weeks of subtle neuron generation might still significantly contribute to single population of cortical GABAergic neurons, especially if this production is directed to specific cortical regions.

Interestingly, some experimental support for this hypothesis is coming from mouse. Riccio et al. (2012) showed the accumulation of proliferating GABAergic progenitors close to the anterior cingulate region in the postnatal mouse. These cells produce calretinin neurons destined for lower cortical layers of anterior cingulate cortex. The proliferation is present until P21 and is notably abundant during first postnatal week. Fate mapping analyses suggest that these neuronal precursors originate from the CGE and LGE.

The first postnatal weeks in rodents are characterized by the completion of migration and onset of intensive differentiation, as well as ingrowth and outgrowth of fibers. These developmental events also characterize last trimester and early postnatal development in primates (Uylings, 2000; Kostovic et al., 2014). If production of calretinin neurons from the GE continues for additional 10–12 weeks after peak of neurogenesis in humans, it might significantly contribute to its proportion. Protracted production of important fraction of cortical GABAergic neurons subpopulation could have large importance in prematurely born infants, where neuron production is altered (Malik et al., 2013). This might lead to neurological and psychiatric disturbances, or alter psycho-motor capability. Regarding incidence of premature deliveries in developing countries, such an event could not be only medical problem but could also have significant social impacts (Petanjek and Kostović, 2012; Raznahan et al., 2012).

## CONCLUDING REMARKS

Calretinin neurons become in primates the most prominent population of cortical GABAergic neurons. Pronounced increase in the number of calretinin neurons is supplied by changes in the developmental rules regarding their origin. Already during the early fetal period, an increased pool of calretinin neuron progenitors is present in the CGE, as well as clear signs of onset of cortical GABAergic neurogenesis. During the middle trimester of gestation, calretinin neurons destined for the frontal cortical regions originate mainly from the frontal cortical proliferative zones and at lower extent from the dLGE. This period is also characterized by the peak of neurogenesis in the GE, where calretinin neurons destined for posterior and lateral cortical regions originate mainly from the CGE. There seems to be ongoing production of calretinin neurons into the last trimester of gestation.

Increased pool of progenitors in the CGE during the early and middle fetal period, abundant cortical production during middle fetal period together with protracted production from the GE during the last trimester of gestation, might explain such an increase in the proportion of calretinin neurons in primates.

## Conflict of Interest Statement

The authors declare that the research was conducted in the absence of any commercial or financial relationships that could be construed as a potential conflict of interest.
